# The Age of Initiation of Drug Use and Sexual Behavior May Influence Subsequent HIV Risk Behavior: A Systematic Review

**DOI:** 10.1155/2013/976035

**Published:** 2013-12-07

**Authors:** Patrick Baldwin, Roman Shrestha, Jessica Potrepka, Michael Copenhaver

**Affiliations:** University of Connecticut, Department of Allied Health Sciences, 358 Mansfield Road, Unit 2101, Storrs, CT 06269-2101, USA

## Abstract

Researchers examining injection drug users (IDUs) in drug treatment have been trying for decades to determine the optimal way to intervene to prevent the transmission and spread of human immunodeficiency virus (HIV) in this population. Although efficacious HIV risk reduction interventions are widely available, questions remain about what specific factors are most related to HIV risk behavior and defined as unprotected sexual activity and/or high risk drug use. This review involved an evaluation of the research literature in order to better understand the association between drug use and sexual behavior debut on HIV risk behavior. Findings suggest that drug use debut and sexual behavior debut may be related to subsequent HIV risk behavior. Evidence to date implies that intervening at an earlier age to assist youth to avoid or delay these high risk behaviors may be an additional means of reducing subsequent HIV risk.

## 1. Introduction

Globally, an estimated 3 million people who inject drugs are living with HIV, representing roughly one in 10 infections worldwide [1]. An estimated 49,273 individuals in the U.S. were infected with HIV in 2011 and injection drug users (IDUs) remain at high risk for HIV transmission. Since the epidemic began, nearly 182,000 injection drug users with an AIDS diagnosis have died, including an estimated 4,218 in 2010. Of the approximate 32,050 new cases of AIDS reported in 2011, 3,961 were IDU-associated. In 2011, 7.4% of all newly diagnosed cases in adults and adolescents were from IDU-associated behavior [2]. With the high rate of HIV risk in the IDU population, it is even more important to understand the events leading to risk behavior.

Part of the global HIV epidemic involves adolescents who are initiating sexual activity at progressively earlier ages [3] and putting themselves and others at increased risk for HIV infection. A 2011 CDC survey found that 47% of high school students reported having sexual intercourse, 6.2% reported having intercourse before age 13, and an alarming 39.8% reported not using a condom during their last intercourse [4]. The average reported age of first sexual intercourse in the USA is age 14.4, with more males than females (9.0% versus 3.4%, resp.), and more African-American teens than other racial/ethnic groups reporting sexual activity before age 13—highlighting a common racial disparity pattern [4, 5]. Although data exist regarding HIV risk and prevalence rates, questions remain about what factors are most predictive of subsequent HIV risk behavior among adolescents and young adults, and thus what strategies could potentially alter this trajectory.

While research has focused on identifying predictors of risk behaviors in adolescence, the influence of the age of debut, defined as the initiation or the first occurrence of certain behaviors such as substance use and coitus, on later HIV risk outcomes remains largely unexplored in the empirical research literature. Health behavior research to date has examined how the debut of specific risk behaviors among youth is predictive of various negative health outcomes among those individuals later in life as adults [6–15]. For example, early sexual debut has been linked to short-term negative outcomes such as higher levels of depression among adolescents [16] and delinquency [17], as well as longer-term negative outcomes including lowered expectations for pursuing higher education [18, 19], poorer academic performance [20], less consistent contraceptive use [21], unintended pregnancies [14], increasingly risky sexual behaviors [22–35], and likelihood of having multiple partners [36–38]. Conversely, other studies indicate that the delay of sexual initiation tends to be protective of future risk behavior [3, 16, 18, 39].

There are clear parallels between sexual debut and substance use debut in terms of the potential pattern of influences on subsequent health risks. A growing number of studies have also been concerned with the linkage between early onset of drug use and a range of negative health risk outcomes that occur in adulthood. For example, an earlier age of substance use debut is a well-documented risk factor for subsequently developing substance use disorders [13, 40–48], poorer academic performance, and lower academic expectations [48, 49]. Drug use debut and HIV transmission risk behavior have also been tentatively linked in several recent studies [50–55], although this linkage was not the primary focus of these studies.

Like other types of substance use in adolescence, drinking alcohol at an early age remains a serious health concern since it may interfere with brain development and lead to cognitive and behavioral dysfunctions that foster risk taking [56] and later substance use problems [6, 57]. Early onset of alcohol consumption may also tend to establish a pattern of maladaptive coping mechanisms in which alcohol is implemented to manage stress [44]. Furthermore, substance use—particularly heavy drug use—during a critical period of neurobiological development may lead to poorer memory and learning functioning, reduced inhibition, abnormalities in executive functioning and neuronal activation, and alterations in brain structure, as well as a host of other neurocognitive sequelae [58].

In addition to the parallels between drug use debut and sexual debut, numerous studies have highlighted a cooccurrence of both behaviors, linking the debut of one type of behavior to the other, or both, and underscore their complex and often interwoven associations [20, 59–64]. For example, one investigation of Swedish high school students found that teens who reported sexual debuts at age 15 used more tobacco, illicit drugs, and alcohol than their aged-matched inexperienced peers, suggesting a clustering of risk behaviors linked with early sexual initiation [65]. By systematically exploring the relevant literature in the present review, we seek to more clearly elucidate the progression of HIV risk behaviors over time so as to better inform future HIV prevention interventions.

## 2. Methods

### 2.1. Selection of Studies

**Figure 1:**
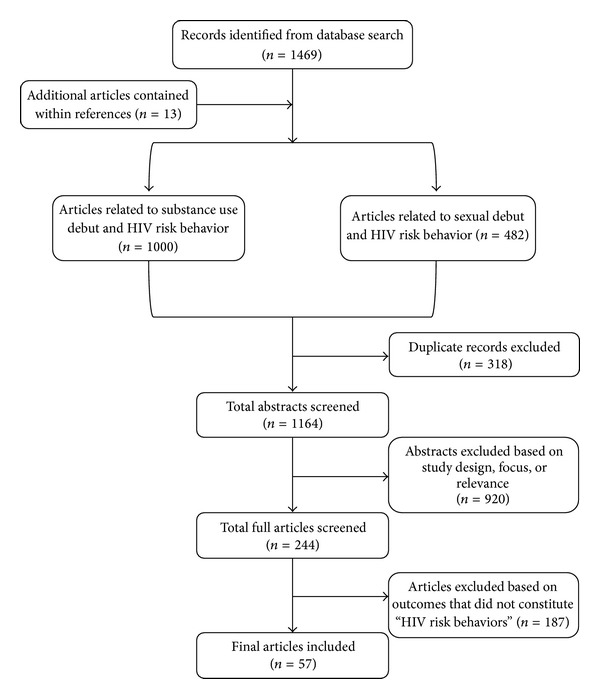
Study inclusion flow diagram.

We searched for studies that included quantitative data about sexual debut, drug use debut, and subsequent HIV risk behavior. In conducting this literature review, peer-reviewed studies were included if they met all of the following criteria: (1) identified either age of sexual debut, age of substance use debut, or both; (2) assessed at least one concurrent or subsequent HIV-related substance use or sexual risk behavior as an outcome; and (3) were published in English (see Figure 1).

### 2.2. Search Strategy

An extensive literature search was conducted in the online database PubMed that included studies from 2001 through December, 2012. Our primary objective was to review scientific data related to the influence of both sexual and substance use debut on subsequent HIV risk behavior. We cross-referenced previous reviews and primary studies for additional citations. In an initial search, both of our domains, drug use debut and sexual debut, were crossed one-at-a-time with consequential HIV risk behavior, using multiple combinations of search terms; see Table 1.

The search was not limited to any particular geographic area or region and there were no restrictions imposed on the age of subject populations. As such, definitions of “early debut” of either risk behavior varied across studies, making preadolescence, adolescence, and young adulthood more useful terms that would ensure coverage of all target groups and encompass a broader age range. Sexual debut was defined as first oral, anal, or vaginal sex. Substance use included all illicit drugs, cannabis, alcohol, and tobacco. HIV risk behavior outcomes included unprotected sexual activities, sexual activities under the influence of drugs and/or alcohol, sex with multiple partners, exchange sex, injection drug use (IDU)—particularly using uncleaned syringes or sharing injection equipment, sex with an IDU partner or partners, STI, pregnancy, and HIV serostatus.

A total of 1,469 articles were retrieved as of December 30, 2012. Thirteen additional articles were found in the reference section of the relevant journal articles. After the removal of 318 duplicated records, 1,164 articles remained for preliminary review. After inspecting study titles and abstracts, we found that 920 records were not directly relevant to the study objectives, leaving 244 records for further, more detailed, review. A full-text copy of this subset of studies was obtained and assessed for inclusion. 187 full-text records were excluded because they did not investigate an association between the variables of interest and behavioral outcomes that constitute HIV risk behaviors. Finally, a total of 57 studies were included for this review (see Figure 1).

## 3. Findings

### 3.1. Early Initiation of Sexual Behavior and Concurrent Health Risks

Early initiation of sexual behavior is viewed as a health risk since youth who initiate sexual behavior at an earlier age spend relatively more years at potential risk for HIV and a range of other sexually transmitted diseases versus those who are not sexually active until a later point. One study found that among a sample of female high school students in Nova Scotia, aged 15 to 19, having vaginal intercourse before 15 years was associated with sexual risk-taking behaviours—such as not using a condom, unplanned intercourse due to substance use, having a casual partner, and having multiple sexual partners—at later points in adolescence [78]. Similarly, Pettifor et al. evaluated risk factors related to early coitarche among 7,692 South African youth and found that 18% of males and 8% of females reported sexual debut at age 14 or younger [91], while a cross-sectional study of homeless 13- to 17-year olds in Brazil revealed that one-third of females and two-thirds of males in the sample reported sexual debuts under the age of twelve [68]. An earlier sexual debut was associated with inconsistent condom use [68] and a lack of condom use [91].

Nkansah-Amankra et al. examined data from the Colorado Youth Risk Behavior Surveillance System (2005)—sampling 1,498 adolescents in 29 public schools, grades 9–12—and found an association between patterns of adolescent drug use and high risk sexual behavior [85]. Females in the sample who reported using two illegal substances were 12 times more likely to report early sexual debuts than those reporting no use, while females using three substances were 44 times more likely to report early sexual debuts. Additionally, adolescent binge drinking was also associated with higher odds of having multiple partners. The results suggested that adolescents with drug-use experience had a greater tendency to report being involved in high risk sexual activity and consequently to be at greater risk for contracting and spreading HIV.

In a seven-year cohort study of American Indian teens aged 14–18 living in a Northern Plains community in South Dakota, Mitchell et al. (2007) found that while predictors of early sexual initiation differed by gender, having used alcohol or drugs at sexual debut was a significant predictor of early sexual initiation for both males and females [82] as other researchers have found [20, 62]. Still another investigation showed that young men in rural South Africa reporting sexual debut before age 15 were more likely to not use condoms at first sex and to have had multiple and casual partners than those who had initiated sex at 15 or older [28]. Likewise, a later investigation revealed that sexual debuters aged 16 and younger, particularly males reporting female partners, were more likely to have had multiple sexual partners than later debuters—highlighting a similar, albeit gender-specific, combination of risky behaviors occurring at sexual debut [35].

Thus, there is evidence from diverse samples that, not surprisingly, earlier initiation of sexual behavior among youth is linked with higher levels of concurrent health risk behaviors among these samples. A similar pattern of findings have been identified with regard to health risks associated with earlier initiation of drug use behavior among youth, as detailed below.

### 3.2. Initiation of Substance Use and Concurrent Health Risks

Miller et al. explored factors associated with IDU initiation before age 16 among 542 IDUs [81]. Thirty-eight percent of the sample initiated IDU prior to age 16. There was also an association between earlier initiation of injection drug use and the following variables: gender (being female), identifying as a sex worker, using other drugs, and having a history of criminal justice system involvement. Early drinkers more likely to report subsequent alcohol problems, unprotected sexual intercourse, multiple partners, and being drunk or high during sexual intercourse. Likewise, a study conducted in Canada assessed the circumstances surrounding the initiation of injection drug use among 560 street-recruited youth (median age of drug use debut was age 13; median age of IDU debut was age 17) and found a significant association between age of first drug use and history of IDU [74]. A study conducted in Russia assessed 558 IDUs and their non-IDUs sexual partners and found that the initiation of alcohol use at a younger age (mean = 14.2 years) was related to an earlier onset of coitarche (mean = 15.5 years) and a younger age of first drug use (mean = 16.8 years) [6]. Thus, a significant correlation was found between age of first alcohol use and the age at first sexual intercourse as well as the likelihood for having multiple sexual partners and age of initiating injection drug use behavior.

A cross-sectional behavioral survey that evaluated 805 street-based adolescents in the Ukraine reported a pattern of elevated HIV risk stemming from both unprotected sex and injection drug use [100]. Almost 75% of the sample of adolescents had experienced sexual behavior debut before the age of 15. Among those who reported injecting drugs within the past month, 44% had shared needles at least once and reported lower levels of condom use. These studies built on earlier studies in this area by examining health outcomes linked with injection drug use initiation among youth from diverse samples.

Noninjection drug use also shares a strong linkage with early coital debut across a vast ethnic and national landscape. Using data gathered from seven national and three local surveys, the National Campaign to Prevent Teen Pregnancy's Research Task Force found that, compared to delayed sexual activity, early sexual activity among young US teens has been linked to a greater number of sexual partners over time. In addition, these surveys found that sexually experienced youth aged 14 and younger are much more likely to smoke, use drugs and alcohol, and participate in delinquent activities than their inexperienced peers [101], though the precise sequence of behaviors remains uncertain. Other national survey data gathered in the USA show that girls who had begun smoking marijuana by age 14 or cigarettes by age 12 were significantly more likely to be pregnant by age 15, though it is plausible that these predictors were moderated by some common underlying risk factors [66]. In a cross-sectional sample of 15- to 21-year-old male and female vocational students in Thailand, recent cigarette smoking, alcohol consumption, and illicit substance use were all strongly associated with earlier onset of sexual intercourse for males, though whether or not experimentation with drugs predisposed these individuals to engage in risky sexual behavior was unclear [79]. Additionally, a 2009 survey assessing risk behavior of Mississippi public high school students [37] found similar associations between drinking alcohol, smoking cigarettes, and using marijuana or other drugs and early sexual initiation. Black respondents, it was found, were more likely to report early sexual initiation.

### 3.3. Debut of Risk Behaviors and Subsequent HIV Risk Outcomes

Although the majority of studies examined associations among concurrent health risk variables, we also identified an expanding body of relevant literature that focused on the influence of early risk behaviors (e.g., sexual activity and drug use) on HIV risk behaviors at later points in life. In addition to the lingering effects of early sexual experiences on a wide range of health outcomes discussed earlier [33], early coitus has an association with HIV risk behaviors, specifically, later in life in a range of diverse contexts and populations.

In a large population-based study of over 23,000 Danish men aged 18–45, Buttmann et al. found that those with early sexual debuts (i.e., sexual activity before age 14) were more likely to have subsequently engaged in risky sexual behaviour (i.e., having 8 or more lifetime sexual partners and two or more current partners) than older debuters [22]. This association was also evident in a cross-sectional survey of 15–18-year-old adolescent boys in Tehran; of those who were sexually experienced, about 15% had sexual debuts by age 12, 35% by age 14, and 55% by age 15. Moreover, early sexual debut was a predictor of multiple sexual partners in one's lifetime. A similar pattern was shown in a 3-year longitudinal study of male and female urban minority adolescents in the USA; early initiators (those who had engaged in sex by 7th grade) were more likely than later initiators (those who had abstained by the end of middle school) have reported multiple sex partners, being involved in a pregnancy, forcing a partner to have sex, having frequent intercourse, and having sex while intoxicated or under the influence [30]. The same study, however, noted significant gender differences in these outcomes; early female initiators were four times more likely to be involved in a pregnancy and were half as likely to report consistent condom use versus their male counterparts, while males were more likely than females to report having had at least four sexual partners.

The association between early sexual debut and subsequent risky behavior or HIV infection is also seen in studies with exclusively female samples [70, 75, 97]. One population-based study of nearly 65,000 women in four Nordic countries illustrated that young age of first sexual intercourse was highly associated with reporting risk-taking behaviors. Among the sample of women aged 18 to 45, young age of first intercourse (age 14 or younger) was highly associated with risk-taking behaviors, such as a greater number of lifetime sexual partners, recent partners, and history of STIs [31].

Fatusi and Wang investigated the association between early coital debut and STIs among a nationally representative sample of 15- to 24-year-old males in Nigeria and found that early sexual debut (sexual intercourse before age 16) had a significant independent association with self-reported STIs [26]. In addition, early sexual initiators were significantly more likely to have sex with casual or commercial partners and multiple partners and less likely to use condoms. Having multiple sexual partners, in this case, mediated the association between age of sexual debut and subsequent STI [26]. Similar findings that clearly show a strong association between early sexual debut and subsequent STIs have also been documented in mixed-gender samples [77] and among gay men [29], while the early sexual debut and multiple partners correlation has been shown among South African youth [35]. Another study of 15- to 24-year-old males in South Africa reported findings consistent with the following: those who initiated sex before age 15 were more likely to report no condom use and a casual partner at first sex. Furthermore, early sexual debut was strongly associated with having more than three partners within the past three years, thus placing them at higher risk for HIV and other STIs [28].

In one study, Fuller et al. focused on both sexual and IDU debut behavior and examined the factors associated with injection initiation during adolescence (i.e., age 21 or younger) compared with young adulthood (i.e., older than age 21) among 226 street-recruited IDUs in the Baltimore, MD, area [72]. The overall median age of IDU initiation was age 23 and 40% of the sample reported initiating IDU during adolescence. Those who reported initiating injection drug use during adolescence tended to demonstrate relatively rapid progression to IDU compared to those who initiated injection drug use as adults. Forty-two percent of study participants reported that their sexual debut occurred prior to age fourteen. Those who reported initiating IDU during adolescence were more likely to report having their first sexual experience prior to age 14 as well as having multiple sex partners in the previous six months [72].

In a later study, Fuller et al. evaluated predictors of age of injection drug use initiation and HIV-related risk behaviors among a similar population—144 IDUs in Baltimore, MD [61]. This study reported that race, neighborhood, and educational level were significant predictors of age of injection drug use initiation. In this study, the median age at initiation of IDU was age 21. Fifty-one percent of the participants had initiated IDU during adolescence. Adolescent initiators were twice as likely as young adult initiators to report that their first sexual experience have taken place before they were 14 years of age. Adolescent-initiating IDUs were less likely than adult-initiating IDUs to report high-risk sex and injection behaviors, but more likely to report high-risk networks. Cheng et al. explored risk factors associated with the initiation of injection drug use among 2,231 injectors in Northern Thailand [45]. The analyses indicated that the age of drug initiation was negatively associated with the risk of injection initiation such that the older the age of initiation of any type of drug use, the lower the risk of initiating injection drug use. Likewise, in a study of IDUS in southern Thailand, HIV status was also correlated with an earlier age of injection initiation [90, 95] and injecting at drug debut [90]—underscoring the importance of drug use debut as a salient HIV risk factor.

Muga and colleagues assessed HIV-related outcomes among 1,111 IDUs in Spain and reported that both age of drug use debut and duration of injection drug use were associated with participants contracting HIV [84]. Subramaniam and Stitzer evaluated adolescents (aged 14–18) who reported substance abuse and their subsequent risk for HIV [93]. Approximately half of injection heroin users in the group reported sharing needles or “works” during this particular period of time, leaving them highly susceptible to Hepatitis-C and HIV infection. Forty percent of the total sample population, however, including both opioid- and heroin-users, engaged in HIV risk behaviors such as having multiple sexual partners and always having unprotected sex. Likewise, Parviz et al. focused on drug- and sex-related risk behavior outcomes among 242 IDUs in Pakistan and found that a slightly younger age of first injection (age 25 versus age 28) was associated with subsequent drug-related HIV risk behavior (i.e., receptive needle sharing) [88]. Forty-seven percent of those who participated in the study engaged in receptive needle sharing, 22% had sexual intercourse—of whom only 7% used condoms—and none had cleaned their needles with bleach.

More recently, Dillon et al. explored the associations among age of sexual behavior debut and sexual risk behaviors among 158 predominantly Latina immigrants (mean age: 27.2 years) living in Miami, FL. The following variables were associated with a younger age of sexual behavior debut: a greater number of sexual partners, more frequent sex-related alcohol or drug use, and greater levels of intoxication from alcohol or drugs during sex [25]. Dorjgochoo et al. also examined the associations between various behavioral factors and HIV status among 3,391 sexually active Haitians [69]. The mean age of sexual debut was age 16.5 for females and age 14.6 for males. HIV infection for female participants was associated with years of sexual activity, confirmed/suspected STI in females, and suspected STI in partners or suspicion that partners had outside sexual partners; HIV infection among male participants was associated with drug use and sexual debut with an unknown person. Interestingly, later sexual debut was not protective of HIV infection among women or men in this sample. Moreover, sexual debut after age 16 was positively associated with HIV infection in males. In contrast, Medhi et al. focused on factors associated with HIV serostatus among 426 female sex workers (mean age of 25.7 years) in India and found in a multivariate analysis that HIV-infected status was significantly associated with lifetime injection drug use, initiating sexual intercourse before age 15 and testing positive for one or more STIs [80]. Likewise, Gore-Felton et al. showed in a sample of 16- to 25-year-old male and female IDUs in Russia (mean sexual debut ages 14.84 and 15.50, resp.) that young age of sexual initiation was significantly associated with sexual risk behaviors in the past 30 days for men but not women [73]. This suggests a strong association between young coital debut and subsequent risky sexual and injection drug use behaviors. Taken together, studies from diverse geographic locations and populations suggest that earlier risk behaviors—including sexual activity and drug use—are significantly associated with HIV risk behaviors at later points in life.

## 4. Discussion

We reviewed studies that included data on the initiation of sexual and drug use behavior and concurrent and subsequent health risk behaviors. Although the majority of studies were cross-sectional and included information linking recent risk behaviors among youth, we also uncovered evidence of a linkage between early initiation of various risk behaviors and health risk behaviors later in life. Not surprisingly, the pattern of outcomes tend to show that the early initiation of sexual behavior and substance use are related to a range of other recent health risks, such as various STIs [26, 27, 33, 67], HIV [75], SUDs such as cannabis use disorders and alcohol use disorders [60, 102], psychiatric disorders including MDD [8], Hepatitis-C and cervical cancer [24], engaging in commercial sex exchange work [26, 71], poor school performance and conduct disorder [52], physical violence [65], receptive needle sharing [86], pregnancy [27, 66], and externalizing disorders [42]. In contrast, the longitudinal associations tend to be less clear cut based on the studies currently available.

The associations between sexual and drug debuts with contemporaneously occurring risk behaviors, as well as those occurring at later points in life, appear to be complex and therefore difficult to precisely capture. In the studies reported to date, it is also difficult to pinpoint the influence of sexual and substance use debuts as other risk factors—that particularly the initiation of drug and/or alcohol use—are often reported to occur concurrently. As indicated above, an early age of sexual initiation is associated with subsequent sexual risk behaviors, but also with subsequent risky drug use behavior [80] and substance use in general [42, 60, 103, 104]. Importantly, both types of risk behavior debuts have been linked to HIV infection [29, 69, 70, 75, 80, 81, 84, 90, 95, 97]. Likewise, the association of an early drug use debut and later risk behavior appears to be multidirectional, as subsequent risky substance use practices (e.g., needle sharing), sexual practices (e.g., multiple partners, transactional sex, lack of condom use), and substance use in general have all been linked. This crossover of risk behavior domains, thus, suggests that early initiation of either type of risk behavior can be associated with the other [6, 24, 25, 27, 31, 37, 49, 63–65, 76, 78–80, 89, 98, 103, 104], and both might operate synergistically (e.g., drinking or illicit drug use coupled with unprotected sex [105]) as part of a tendency toward a generally riskier lifestyle.

The overall influence of earlier initiation of sexual activity tends to be negative but varies significantly across ethnicities and/or geographic location, socioeconomic status, gender [30], and sexual orientation, thus precluding universal generalizations, as might be expected. Despite disagreement among researchers about whether early sexual involvement fits into a larger constellation of generic “problem behaviors” or merely experimentation as a normative part of development [33, 106, 107], theories suggest that sensation-seeking [63, 105], increased feelings of invulnerability, and risk-proneness—or a propensity to be attracted to potentially risky behavior—likely play prominent roles in influencing early sexual debuts [14]. Adolescence, after all, is a period preceding full cognitive development and emotional maturity [108].

The larger cultural contexts in which these behaviors occur must be considered, as sexual and substance use norms and perceptions are often shaped by religious, national/regional, and social values. In many cultures, adolescent sexual activity is increasingly viewed as a normative aspect of developing responsible intimate relationships as teens mature into adulthood, rather than a delinquent behavior like drug use [30, 33, 107]. Consequently, the disparate cultural norms inherent in the vast array of countries, communities, and ethnic subgroups included in this review may be expected to differentially influence the trajectories of subsequent risk behaviors [62].

The age of sexual initiation carries serious implications for STI risk; early sexual debut potentially increases the duration of lifetime sexual exposure, allowing a relatively longer sexual career overall and, hence, more time for individuals with young debuts to accrue sexual partners, especially during adolescence, when relationships are often short in duration and changing sexual partners is more common [22, 26, 31] and risk-proneness is heightened [21]. The importance of considering the nature of and context in which initial sexual experiences occur cannot be overstated, as sexual coercion, sex under the influence, and rape are profoundly different sexual experiences than consensual intercourse within the context of a trusting, intimate relationship, for instance, and have profoundly different implications for influencing future risk behavior [32, 99]. Many studies simply were not designed to disentangle these important qualitative details nor the emotional context surrounding diverse experiences surrounding the first sexual intercourse.

A similar rationale has been proffered to explain the link between early substance initiation and IDU behavior later in life. Earlier exposure and access to substances during a period of psychosocial development, marked by relatively rapid cognitive changes and strong peer influence, create a greater window of potential risk [94]. Additionally, poorer performance on executive functioning tasks among adolescent substance users suggest a reduced ability to control behavior, make healthy long-term decisions, and attend to environmental stimuli which may put individuals at greater risk for substance use later in life [58, 94]. Thus, considered as a whole, studies to date indicate that early initiation of sex- and drug-related behaviors may exert substantial influence on youth from diverse backgrounds and geographical regions and may set them on a different HIV risk trajectory relative to their counterparts who do not have an early debut of such behaviors.

### 4.1. Study Limitations

Some of the limitations of this systematic review should be acknowledged. First, the review was restricted to peer-reviewed journal articles published in English, which likely biased our collection toward primarily English-speaking countries. The search itself was restricted to one database, although this is unlikely to have been a major limitation as PubMed Central (PMC), with nearly 2.4 million biomedical articles, is the largest repository of full-text open-access biomedical articles in the world. In addition, only abstracts were screened for this review to determine whether the study investigated the impact of early sexual and substance use debuts on HIV risk behavior outcomes. Thus, any secondary findings and analyses relevant to our topics of interest not mentioned within the abstracts may have been excluded from this review. Many behaviors were self-reported, often at much later time points, making response bias and social desirability potential limiting factors.

We should also add that, due to a reliance on cross-sectional designs in many of the studies included, it is not possible to establish causal inferences and, hence, precludes any definitive interpretations regarding the processes by which sexual and substance use debuts independently lead to later risky behaviors or the precise sequence of their trajectories. Since certain antecedents and common underlying factors, such as PTSD and CSA (childhood sexual abuse), parental substance use history, peer influence, and the role of genetic predispositions to substance dependence were not the foci of most of the studies included in this literature review, disentangling these potential confounders was well beyond the scope of our review.

### 4.2. Future Directions

Despite the noted limitations, the findings of this study have implications for future interventions and HIV risk prevention programs. Of the Centers for Disease Control and Prevention's (CDC) 18 evidence-based HIV Risk Prevention Interventions targeting “high-risk youth,” eight address both drug and sexual risk behaviors. Of those, however, five are designed for either nonsubstance dependent or precoital adolescents, and only two of which meet the CDC's best evidence criteria of showing empirical evidence that demonstrates a reduction in HIV/STD incidence, reduced HIV-related risk behaviors, reduction in HIV viral load, or improvement in HIV medication adherence behaviors. Thus, a need exists for innovative EBIs that optimally target high risk youth in a manner that prevents or disrupts the linkage between the initiation of sex- and drug-related behaviors and subsequent HIV risk. Screening adolescents for risk factors may also prove a potent preventative measure given the established association between early debuts of substance use, sexual intercourse, or both, as it could potentially obviate later secondary and tertiary prevention efforts.

Interventions that cross multiple domains of risk (i.e., focus on delaying the initiations of sexual behaviors and substance use and reducing harm following initiation) may have a larger impact than addressing either one by itself in reducing the risk for STD transmission, pregnancy, initiation of IDU, and HIV infection. Interventions are needed that focus on the associations between substance use, multiple sexual partners, failure to use condoms, and HIV infection and that stress the potential harms of alcohol and other substances on judgment and risk taking. Secondly, interventions should confront attitudes about condom use and sexual norms in a culturally competent and relevant manner—tailoring messages to fit within the target audience's larger religious, social, and cultural contexts [31, 62, 83]. A “one size fits all” approach would overlook the diverse findings of the studies reviewed that vary among different ethnic groups and communities of disparate socioeconomic statuses. Environmental context, race, sexual orientation [29, 87], and gender must all be considered when designing behavioral interventions targeting adolescents [61].

Lastly, future prevention interventions should target younger, precoital adolescents in late elementary and early middle school who are cognitively susceptible to experimenting with a range of risk-taking behaviors. Early screening to assess for behaviors such as drinking and cigarette smoking might help to quickly identify those beginning to experiment and assist them in averting a trajectory with potentially deleterious health outcomes. Similarly, an efficacious assessment tool could provide an early warning sign to parents, school counselors, medical professionals, and other professionals in positions to provide proper education and counseling regarding the short- and long-term health consequences of initiating risky behaviors during adolescence.

## Figures and Tables

**Table 1:** Search terms and terminology used for retrieval of eligible citations and reports.

“coital” **AND** “debut” **AND** “risk”		
“substance” **AND** “debut” **AND** “HIV”		
“risk behavior” **AND** “initiation” **AND **“subsequent”		
“drug initiation” **OR** “sexual initiation” **AND** “risk” **AND** “HIV”		
“Substance use” **OR** “drug use” **OR** “needle sharing” **OR** “syringe sharing” **OR** “first injection” **OR** “drug initiation” **OR ** “IDU”	**AND**	“age of initiation” **OR** “age of debut” **OR** “age of first use” **OR** “early initiation” **OR **“early age” **OR** “age of onset”
“Sexual debut” **OR** “sexual intercourse” **OR** “sexual initiation” **OR** “risky sexual behavior”	**AND**	“subsequent” **OR** “early initiation” **OR** “adolescence” **OR** “early age” **OR** “age of onset” **OR** “age of first use”

**Table 2:** 

Study	Study location	Sample size (*N*) (sex; age)	Study design	Study population	Summary of findings
Abdala et al., 2012 [6]	St. Petersburg, Russia	*N* = 558 (M,F; 18–58)	Cross-sectional	IDUs and non-IDUs sexual partners of IDUs (participants of SATH-CAP study)	Age at first drink affects multiple sex partners through age at sexual debut and injection drug use, but no effect on unprotected sex; age at first drug use not related to sexual risk behaviors.
Buttmann et al., 2011 [22]	Denmark	*N* = 23,080 (M; 18–45)	Cross-sectional	Male residents of Denmark	Men who had sex with a commercial sex worker and those who reported >8 lifetime partners or ≥2 recent sex partners were more likely to have other risk taking behaviors. Men who started sexual activity at a young age, defined as ≤14 years, were also more likely to report >8 sexual partners.
Cavazos-Rehg et al., 2011 [59]	USA	*N* = 13,580 (M,F; 17 years and older)	Cross-sectional	High school seniors (participants in the 1999–2007 YRBS survey)	Severity of substance use was more closely related to higher number of sexual partners than age of substance use onset.
Cavazos-Rehg et al., 2012 [66]	USA	*N* = 8,219 (F; 15 years and younger)	Cross-sectional	Adolescent girls (participants in the 1999–2003 YRBS survey)	Pregnancy linked with substance use onset; of girls pregnant by age 15 years 16% had smoked marijuana by age 10 years and >20% had smoked cigarettes and initiated alcohol use by age 10 years and marijuana use by age 14 years.
Celentano et al., 2008 [67]	Chiang Mai, Thailand	*N* = 658 (M,F; 18–25)	Cross-sectional	Sexually active young adults	Men reported greater number of sexual partners than women, infrequent condom use at last sex; women ≥20 years of age, with ≥2 heterosexual partners in the past year and a younger age at sexual debut, were significantly more likely to have a prevalent STI.
Cheng et al., 2006 [45]	Chiang Mai Province, Thailand	2,231 (M,F; 12 years and older)	Cross-sectional	Opiate- or methamphetamine-dependent individuals receiving inpatient treatment	Age of drug initiation and having multiple sex partners were both associated with increased risk of injection initiation.
Cooper et al., 2007 [24]	Cape Town, South Africa	*N* = 2,065 (F; <60 years)	Case-control	Women (resident of Cape Town)	Early sexual debut associated with increased number of lifetime partners and alcohol use; number of sexual partners associated with sexual debut.
Cornelius et al., 2007 [60]	Pittsburgh, USA	*N* = 136 (M; 10–12)	Cohort	Adolescents (from high risk families based on fathers' SUD)	Earlier age at first intercourse and deviant activities of peers each predicted a significantly higher risk of subsequently developing substance use disorders (SUD).
de Carvalho et al., 2006 [68]	Porto Alegre, Brazil	*N* = 176 (M,F; 10–18)	Cross-sectional	Children and youth in street circumstances	Correlates of unsafe sex included younger age of sexual debut and having a steady sex partner; correlates of illicit drug use included lack of family contact, increased hours in the street daily, having had an HIV test, and older age.
Dillon et al., 2010 [25]	Florida, USA	*N* = 316 (F)	Cross-sectional	Latina mother-daughter dyads (living in urban area)	Having a mother who used drugs during the participants' childhood or adolescence was significantly related to age of sexual debut; younger ages of sexual debut were associated with drug abuse and more sexual risk behaviors.
Dorjgochoo et al., 2009 [69]	Port-au-Prince, Haiti	3,391 (M,F; 13–25)	Cross-sectional	Young adults and adolescents receiving voluntary HIV testing	HIV was associated with drug use and sexual debut with a casual/unknown person among males and suspected/confirmed STI and years of sexual activity among females. Among males, sexual debut >16 years old was positively associated with HIV-infection. Later sexual debut was not protective from HIV in women or men.
Drain et al., 2004 [70]	122 developing countries	(M,F)	Cross-sectional (ecologic/population-level analysis)	HIV seroprevalences per 100 adults 15–49 years old and number of children (younger than 15 years of age) living with HIV infection for the beginning of the year 2000 from the Joint United Nations Programme on HIV	Countries with earlier ages at first sex, higher teenage birth rates, and higher fertility rates had higher HIV seroprevalence.
Duong et al., 2008 [71]	Hai Phong, Vietnam	*N* = 643 (M,F; 18–29)	Cross-sectional	Young men and women	Early age at first intercourse was associated with having sex with a sex worker.
Fatusi and Wang, 2009 [26]	Nigeria	*N* = 1,278 (M; 15–24)	Cross-sectional	Sexually experienced never-married young males	Early sexual debut was associated with STIs; multiple sexual partnership is a mediator of the association between early debut and STI.
Fuller et al., 2001 [72]	Maryland, USA	*N* = 226 (M,F; 15–30)	Cross-sectional	Street IDUs (adolescence versus young adulthood)	Factors significantly associated with adolescent initiation of IDU included race other than African American and practices prior to initiating injection including condom use, lack of cocaine use, exclusive crack smoking just prior to initiation, and smoking marijuana.
Fuller et al., 2005 [61]	Maryland, USA	*N* = 144 (M,F; 15–30)	Cross-sectional	Street IDUs with ≤5 years of injection use	Adolescent-initiating IDUs were less likely than adult-initiating IDUs to report high-risk sex and injection behaviors and more likely to report high-risk networks. African American IDUs from neighborhoods with large percentages of minority residents and low adult educational levels were more likely to initiate injection during adolescence than White IDUs from neighborhoods with low percentages of minority residents and high adult education levels.
Gore-Felton et al., 2003 [73]	St. Petersburg, Russia	*N* = 188 (M,F; 14–25)	Cross-sectional	Young adult IDUs	Males who initiate sex at a younger age are more likely to report multiple sex partners.
Hadland et al., 2010 [74]	Vancouver, Canada	*N* = 560 (M,F; 14–26)	Cross-sectional	Street youth (participants of At Risk Youth Study)	Youth previously injected were more likely to have engaged in noninjection use of heroin or of crystal methamphetamine.
Hallett et al., 2007 [75]	Rural Zimbabwe	*N* = 9,086 (M,F; 17–54, 15–44; resp.)	Cross-sectional	Household couple (Zimbabwe household census)	Age at first sex declined among males over the past 30 years but increased recently among females; early sexual debut before marriage precedes a lifetime of greater sexual activity but with more consistent condom use; women who begin to have sex earlier than others of their age are more likely to be infected with HIV.
Hansen et al., 2010 [27]	Denmark, Iceland, Norway, and Sweden	*N* = 69,486 (F; 18–45)	Cross-sectional	Women who initiated smoking early (before age 15) and later (at 15 or later) and never smokers	Adult women who initiated smoking early reported more lifetime and recent sexual partners and less condom use and had lower debut ages for coitus, pregnancy, and alcohol consumption.
Harrison et al., 2005 [28]	Rural South KwaZulu/Natal South Africa	*N* = 314 (M; 15–24)	Cross-sectional	Adults (household census)	Men with sexual debut at less age than 15 were more likely to report risk behaviors at first sexual experience: no condom use (19%), a casual partner (26.8%), and not feeling they had been “ready and wanted to have sex” (19.5%); early sexual debut was strongly associated with >3 partners in the past 3 years.
Jackson et al., 2012 [76]	Glasgow City, UK	*N* = 3,104 (M,F; 15 and 18-19)	Cohort	Adolescents and Young Adults (participants in two cohorts, born 12 years apart)	Strong association between substance use and sexual risk behavior during early and late adolescence; significant between-cohort difference was a stronger association between female early adolescent smoking and early sexual initiation in the later cohort. Also, relationships between illicit drug use and both early sexual initiation and multiple sexual partners in late adolescence were significantly stronger among girls.
Kaestle et al., 2005 [77]	USA	9,844 (M,F; 18–26)	Longitudinal	Adolescents and young adults with STIs in wave 3 of National Longitudinal Study of Adolescent Health	Younger ages at first intercourse were associated with higher odds of STI in comparison with older ages, but the effect diminished with increasing current age. The association between timing of first intercourse and STIs did not vary by gender, race, ethnicity, or parental education.
Langille et al., 2010 [78]	Cape Breton, Nova Scotia, Canada	*N* = 797 (F; 15–19)	Cross-sectional	High school girls	Early vaginal intercourse was associated with not using a condom at last intercourse, unplanned intercourse in the previous year due to substance use, having a casual partner at last intercourse, and having three or more partners for vaginal intercourse in the previous year.
Liu et al., 2006 [79]	Northern Thailand	*N* = 1,725 (M,F; 15–21)	Cross-sectional	Vocational school students	Sexual initiation was associated with using alcohol or methamphetamine; for males, initiation was associated with friend as a confidant, tobacco use, high perceived risk for HIV, and high STI knowledge; for females, other factors associated with earlier initiation were younger age at interview, living away from family, lacking a family member as a confidant, high-perceived risk for STIs, and ever having smoked marijuana.
Lyons et al., 2012 [29]	Australia	*N* = 845 (M; 16–65)	Cross-sectional	Gay men born between 1994 and 1993	HIV-positive men were found to be significantly younger on average when they first had anal intercourse compared with HIV-negative men; men with a history of other STIs were significantly younger; engaging in higher risk sexual behavior was a likely factor, with AFAI generally younger among men who reported >10 sexual partners in the past year and who engaged in group sex and receptive anal intercourse or were drug or alcohol affected during their most recent sexual encounter.
Magnusson et al., 2011 [14]	USA	*N* = 3,588 (F; 15–44)	Cross-sectional	Fertile women (participants of the 2006–2008 National Survey of Family Growth)	Compared with women who were 18 or older at first intercourse, women who were <15 years of age at the time of first intercourse were nearly two times as likely to report a gap in contraceptive use.
McGuire et al., 2012 [37]	Mississippi, USA	(M,F)	Cross-sectional	High school students (participants of the 2009 Mississippi Youth Risk Behavior Survey)	Older age, being a black, drinking alcohol, or using marijuana or other drugs were associated with early sexual initiation and having multiple sexual partners; heavy smoking was associated with early sexual initiation.
Medhi et al., 2012 [80]	Nagaland, India	*N* = 426 (F; ≥18 years)	Cross-sectional	FSWs (participants in Integrated Biological Behavioural Assessment study)	Initiating sexual intercourse before the age of 15, more than 2 years of sex work, testing positive for more than 1 STIs, lifetime IDU history, exchange sex for drugs, and having IDU sexual partners were all associated with HIV seropositivity. Sexual debut <15 was independently associated with HIV.
Miller et al., 2006 [81]	Vancouver, Canada	*N* = 542 (M,F; ≤16 years and ≤ 25 years)	Cohort	IDUs (participants of the Vancouver Injection Drug Users Study)	Proportion of young initiators was greater among, females, sex workers, binge drug users, and those who have been in juvenile detention or jail; early initiates were more likely to be infected with HIV and hepatitis C virus.
Mitchell et al., 2007 [82]	South Dakota, USA	*N* = 474 (M,F; 14–18)	Cross-sectional	Cohorts of American Indian youths (participants of The Voices of Indian Teens (VOICES, 1993–1995; Waves 1–3) and Pathways of Choices (CHOICES, 1996–1999; Waves 4–7)	For young men, younger initiation of sex was predicted by a greater likelihood of having used alcohol or drugs at first sex; for young women, earlier initiation was related to having mothers who had their first child at an early age and using alcohol/drugs at first sex; higher cumulative risk was associated with elevated risk of sexual initiation.
Mohammad et al., 2007 [83]	Tehran, Iran	*N* = 1,385 (M; 15–18)	Cross-sectional	Unmarried adolescent males	Having no access to Internet, feeling regretful at sexual debut, having one sexual partner in lifetime, and lower knowledge of condoms predictors of condom nonuse; older age, using alcoholic drinks, early sexual debut, and poor knowledge of reproductive physiology are predictors of multiple sexual partners.
Muga et al., 2003 [84]	Barcelona, Spain	1,111 (M,F)	Cross-sectional	IDUs admitted to hospital detoxification unit	Age at start of and duration of injection drug use were associated with HIV infection (*P* < .001).
Nkansah-Amankra et al., 2011 [85]	Colorado, USA	1,498 (M,F)	Cross-sectional	Public high school students	Female teens using 2 illicit substances were 12 times more likely to report early debuts than abstinent teens; females using 3 illicit substances were 44 times more likely to report early debuts.
Novelli et al., 2005 [86]	Maryland, USA	*N* = 420 (M,F; 15–30)	Cross-sectional	Young urban IDUs (participants of the REACH III study)	Adjusting for race, gender, and homelessness, following variables independently associated with recent receptive syringe sharing: age at first hit, self-injection at initiation, and using a syringe that had previously been used by someone else at first hit.
O'Donnell et al., 2001 [30]	New York, USA	*N* = 1,287 (M,F)	Cross-sectional	Urban minority adolescents in 7th and 8th grade.	Early initiators had an increased likelihood of having had multiple sex partners, forced a partner to have sex, had frequent intercourse, and had sex while drunk or high.
Olesen et al., 2012 [31]	Denmark, Iceland, Norway, and Sweden	*N* = 64,659 (F; 18–45)	Cross-sectional	Women in four Nordic countries	OR of reporting >10 lifetime sexual partners was almost four times higher among women who reported a young age at first intercourse; women who were young at first intercourse were more likely to report two or more recent partners and to have a history of STIs; young age at first intercourse was associated with current smoking and binge drinking.
Outlaw et al., 2011 [87]	USA: New York, North Carolina, Michigan, Texas, and California	*N* = 363 (M; 13–24)	Cross-sectional	Non-White MSM	Participants having a MSM sexual debut before the age of 16 reported more exchange sex, drug use, and emotional/psychological problems related to substance use.
Parviz et al., 2006 [88]	Karachi, Pakistan	242 (M)	Cross-sectional	IDUs	Younger age of first injection was associated with subsequent receptive needle sharing. 47% of respondents shared needles and 22% had sexual intercourse—of whom 7% used condoms. No respondents cleaned needles with bleach.
Pechansky et al., 2011 [89]	Porto Alegre, Brazil	*N* = 200 (M,F)	Cross-sectional	Ecstasy and LSD users	Participants with early sexual debut (<14) more likely to report lifetime use of marijuana and powder and crack cocaine; early sexual debut was associated with past year sexual risk behaviors, including having sex while high and having two or more sex partners.
Perngmark et al., 2003 [90]	Southern Thailand	*N* = 272 (M,F)	Cross-sectional	Active IDU	HIV seropositivity among ethnic Thai was independently correlated with past history of needle sharing, injecting immediately at drug onset, and starting first injection at younger age.
Pettifor et al., 2004 [32]	In and around Harare, Zimbabwe	*N* = 4,393 (F; 18–35)	Cross-sectional	Sexually active women (from public sector family planning clinic)	Women with early coital debut had a significantly higher risk profile, including multiple lifetime partners; HIV risk was increased for women reporting early age of coital debut.
Pettifor et al., 2009 [91]	South Africa	*N* = 7,692 (M,F; 15–24)	Cross-sectional	Sexually active youth	Lack of condom use at first sex associated with early coital debut and forced sex for males; among females, the likelihood of nonuse elevated for respondents who had an early debut but had not had forced sex, and among those who had had both a later debut and forced sex.
Prado et al., 2009 [92]	Florida, USA	*N* = 254 (M,F)	Cross-sectional	Hispanic Adolescents of seventh grade year	Larger proportion with high intrapersonal risk for unsafe sex (irrespective of ecodevelopmental risk) report early sex initiation and sexually transmitted disease incidence.
Rothman et al., 2009 [62]	Massachusetts, USA	*N* = 11,110 (M,F; 14–21)	Cross-sectional	Black, Hispanic, and White adolescents	Age at first sex decreased linearly with decreasing age at first drink for all adolescents; significant positive trends between age at first drink and age at first sex were observed for all race and ethnic subgroups.
Sandfort et al., 2008 [33]	San Francisco, USA	*N* = 8,466 (M,F; 18 years and older)	Cross-sectional	US adult population (participants of the 1996 National Sexual Health Survey)	Early initiation of sexual intercourse was associated with various sexual risk factors, whereas late initiation was associated with fewer risk factors.
Santelli et al., 2001 [34]	USA	*N* = 7,441 (M,F; 14–22)	Cross-sectional	Unmarried young people (participants of the 1992 YRBS survey)	Failure to use a condom was strongly associated with the lifetime substance-use scale or, alliteratively, with age at initiation of alcohol.
Stanton et al., 2001 [63]	Maryland, USA	*N* = 383 (M,F; 9–15)	Cohort	African-American adolescents	Early initiators of sex were significantly more likely to report involvement in substance use and drug-delivery/sales.
Stueve and O'Donnell, 2005 [64]	New York, USA	*N* = 1,034 (M,F; 11–14)	Cross-sectional	African American and Hispanic youths at 7th and 8th grade	Early drinking was associated with alcohol and sexual risks through midadolescence; more likely to report subsequent alcohol problems, unprotected sexual intercourse, multiple partners, being drunk or high during sexual intercourse.
Subramaniam and Stitzer, 2009 [93]	Baltimore, Maryland	*N* = 94 (M,F; 14–18)	Cross-sectional	Treatment-seeking OUD adolescents	Roughly 50% injection heroin users reported sharing injection equipment/paraphernalia and 40% of total sample (opioid and heroin users) engaged in HIV-sexual risk behaviors.
Trenz et al., 2012 [94]	Maryland, USA	*N* = 651 (M,F)	Cross-sectional	Adult IDUs and non-IDUs (participants of the NEURO-HIV Epidemiologic Study)	IDUs differed from non-IDUs in age of initiation for cigarettes, marijuana, and alcohol.
Uusküla et al., 2010 [95]	Tallinn, Estonia	*N* = 350 (M,F; 18 years or older)	Cross-sectional	IDUs	Individual with earlier IDU initiation, injecting opioids, and receptive syringe sharing as significant predictors of individual HIV-positive status.
Vicknasingam et al., 2009 [96]	Malaysia	*N* = 526 (M,F)	Cross-sectional	IDUs in nontreatment settings	Being 44 years or younger; not holding regular job; initiating drug use at age 23 or younger; being a morphine user; sharing injecting equipment and having multiple-sex partners were all risk factors associated with HIV seropositivity, though only last 2 remained significant in multivariate analysis.
Wand and Ramjee, 2012 [97]	Durban, South Africa	*N* = 3,492 (F; 18–49)	Cross-sectional	Sexually active women (participants of MIRA study)	Lowest quintiles of age at sexual debut, a higher number of lifetime sexual partners, and being diagnosed as having STIs were significantly associated with prevalent HIV infection.
Wang et al., 2011 [98]	Southern, Midwestern, and Eastern regions of USA	*N* = 7,372 (M,F; 14 years and older)	Cross-sectional	Entering freshmen on HBCU campuses (participants of HBCU Substance Use Survey)	Early sex was modestly associated with subsequent illegal drug initiation, particularly among females.
Yode and LeGrand, 2012 [99]	Burkina Faso, Malawi, and Uganda	*N* = 2,842 (M,F; 14–19)	Cross-sectional	Adolescents (participants of the 2004 National Surveys of Adolescents)	Initiation of sexual activity <14 years of age was more likely to be associated with having a casual sex partner and less likely to be associated with condom use at first sexual relation or with systematic condom use in the past 12 months.
Zuma et al., 2010 [35]	South Africa	*N* = 2,875 (M,F; 15–24)	Cross-sectional	Sexually active youth	Having multiple sexual partners was significantly more common among those that had early sexual debut.

## Appendix

For more details see Table 2.
